# Supplementary material for: Characteristics and visual outcome of ocular trauma patients at Queen Elizabeth Central Hospital in Malawi
Source: PLoS One. 2021 Mar 29;16(3):e0246155. doi: 10.1371/journal.pone.0246155 (PMC8007040; doi:10.1371/journal.pone.0246155)
Supplement: S1 File — (DOCX) [file pone.0246155.s002.docx]

# ENGLISH CONSENT FORM

Patient or Guardian Consent Form

**Pattern and visual outcome of ocular trauma patients treated at Lions sight first eye hospital, Blantyre, Malawi.**

| **Study Number** | **Patient identification Number** |
| --- | --- |
|  |  |

This consent form establishes that you have read and understood what taking part in this research study will involve. Please initial the below boxes to confirm whether you agree or disagree with each of the statements below.

1. I confirm that I have read and understood the information sheet for the above named study and have had the opportunity to ask questions.
2. I understand that taking part is voluntary and that I am free to withdraw at any time, without giving any reason.
3. I understand that any information that I give will only be used anonymously and I will not be identified when study findings are presented in any publications and reports.
4. I agree to take part in this study.
5. I voluntarily agree that my personal information can be collected for the purpose of this study.
6. I agree that my medical documents may be accessed and analysed for purposes of this study.

| **Name of Participant** | **Signature of Participant** | **Date** |
| --- | --- | --- |
|  |  |  |
| **Name of Witness*** | **Signature of Witness*** | **Date*** |
|  |  |  |
| **Name of investigator gaining consent** | **Signature of investigator gaining consent** | **Date** |
|  |  |  |
| ***If participant cannot read or write.** | |  |
