# Supplementary material for: Characteristics and visual outcome of ocular trauma patients at Queen Elizabeth Central Hospital in Malawi
Source: PLoS One. 2021 Mar 29;16(3):e0246155. doi: 10.1371/journal.pone.0246155 (PMC8007040; doi:10.1371/journal.pone.0246155)
Supplement: Attachment — Submitted filename: PLOS One Review.docx [file pone.0246155.s003.docx]

1. It has to be clearly mentioned in the Materials and Methods section as to how patient consent was obtained.
2. The incidence of ocular trauma in children was extrapolated to be 2.3 per 100,000 population. This Reviewer does not believe that the sample size of the study is sufficient to extrapolate the rates to a large population.
3. In Table 2, what constitutes “organic material”?
4. The demographic information about education and occupation does not add any context to the manuscript since the data analysis in Table 5 does not account for injuries based on occupation/education. Table 5 may be improved by including such analysis.
